# hnRNPK knockdown alleviates NLRP3 inflammasome priming by repressing FLIP expression in Raw264.7 macrophages

**DOI:** 10.1080/13510002.2020.1857157

**Published:** 2020-12-03

**Authors:** Junxia Feng, Hongyan Li, Jingchun Li, Ping Meng, Lina Wang, Chunli Liu, Shili Zhao, Wei Sun, Yunfang Zhang

**Affiliations:** a The Central Laboratory, Affiliated Huadu Hospital, Southern Medical University, Guangzhou, People’s Republic of China; b Department of nephrology, Affiliated Huadu Hospital, Southern Medical University, Guangzhou, People’s Republic of China

**Keywords:** hnRNPK, NLRP3 inflammasome, FLIP, macrophages, LPS/ATP, IL-1β, IL-18, chronic kidney disease

## Abstract

**Objectives:** Inflammation is an important predisposing and progressive factor in chronic kidney disease (CKD). Heterogeneous nuclear ribonucleoprotein K (hnRNPK) is associated with many fundamental cellular processes, but in chronic inflammatory pathologies remains unclear.

**Methods:** An *in vitro* peripheral inflammation model was established using lipopolysaccharide (LPS)-stimulated mouse RAW264.7 macrophages, followed by inflammasome activation by ATP treatment. Knockdown of hnRNPK by sihnRNPK and FLICE-like inhibitory protein (FLIP) by siFLIP transfection were achieved in Raw264.7 macrophages. ELISA was used to determine the expression of IL-1β, IL-18 and TNF-α. Real time PCR was applied to detect the mRNA levels of hnRNPK, NOD-like receptors family pyrin domain-containing 3 (NLRP3), FLIP, Caspase-1, IL-1β and IL-18. Western blot and immunofluorescence were performed to detect relevant protein expressions. Co-immunoprecipitation (Co-IP) was used to assess the interaction of hnRNPK with FLIP.

**Results:** Results showed that LPS plus ATP activated NLRP3 inflammasome, which evidenced by the up-regulation of TNF-α, IL-1β and IL-18. Notably, hnRNPK and FLIP were significantly up-regulated in activated NLRP3 inflammasome of macrophages. HnRNPK or FLIP knockdown significantly suppressed the activation of NLRP3 inflammasome, as reflected by down-regulation of Caspase-1, IL-1β and IL-18. Importantly, hnRNPK could directly bind to FLIP in activated NLRP3 inflammasome.

**Discussion:** Our findings suggest that hnRNPK could promote the activation of NLRP3 inflammasome by directly binding FLIP, which might provide potential new therapeutic targets for CKD.

## GRAPHICAL ABSTRACT

**Figure:**
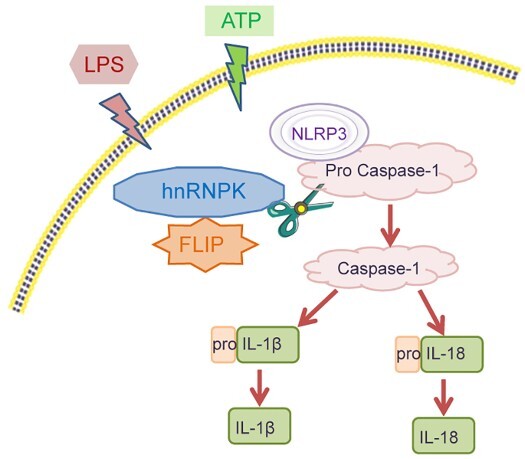

## Introduction

Chronic kidney disease (CKD) is a serious health issue affecting kidney structure and function with an increasing prevalence rate worldwide [1]. Most CKD patients share several risk factors, including hypertension, diabetes, obesity, and metabolic alterations [2–4]. Unfortunately, there is no radical cure for CKD, but preserve the residual kidney as far as possible to delay the progression to end-stage renal failure.

Accumulating studies have shown that inflammatory response is an important predisposing and progressive factor in CKD [5–7]. Inflammasomes, multi-protein cytoplasmic complexes, function as pattern recognition receptors to participate in activation of inflammatory caspases, various immune and cellular death pathways [8,9]. By activating caspase-1, inflammasome cleaves pro-inflammatory cytokines IL-1β and IL-18 to their active and secreted forms [10]. NOD-like receptors family pyrin domain-containing 3 (NLRP3) inflammasome is recently considered to be the best characterized inflammasome, which could trigger caspase-1 activation and inflammation factors release in response to diverse stimuli in CKD patients [5,11]. To date, several studies have demonstrated that elevated levels of NLRP3, caspase-8, IL-1β and IL-18 are critical components of inflammatory process in models of kidney disease [12–15].

Heterogeneous nuclear ribonucleoprotein K (hnRNPK) contains three K homology (KH) domains responsible for DNA/RNA binding and take part in the regulation of gene transcription, RNA splicing and mRNA translation [16] and one K protein interactive region (KI) domain responsible for protein-protein interactions [17], which is one of the most extensively studied hnRNP family members [18]. High expression of hnRNPK is associated with tumor formation, development and prognosis, including colorectal adenocarcinoma [19], oral squamous cell carcinoma [20] and pancreatic cancer [21]. Interestingly, a previous study indicated that hnRNPK could mediate insulin inhibition of renal angiotensinogen gene expression and prevention hypertension and kidney injury in diabetic mice [22]. Another research demonstrated that hnRNPK could promote the activation of macrophages by regulating the lipopolysaccharide (LPS)-induced TAK1 mRNA translation, and up-regulate the transcription of TNF-α, IL-1α, IL-1β and IL-10 [23]. FLICE-like inhibitory protein (FLIP) is found to be critical for protecting rheumatoid arthritis synovial macrophages from Fas-mediated apoptosis [24]. A study from Huang et al. [25] showed that reduction of FLIP in macrophages might be an effective therapeutic approach to suppress inflammation depending upon the disease stage. Additionally, it has been reported that hnRNPK could up-regulate the transcriptional level of cellular FLIP to negatively regulate the tumor necrosis factor (TNF)-related apoptosis-inducing ligand (TRAIL) [26]. Our previous study showed that the protein level of FLIP was positively correlated with hnRNPK. These findings imply that hnRNPK may potentially play a role in chronic inflammatory pathologies by binding FLIP.

Raw264.7 macrophages are commonly used to explore kidney disease related to inflammation *in vitro* [27,28]. As a main component of the cell wall of gram-negative bacteria, LPS widely used to cause inflammatory response *in vitro* and *in vivo*, including kidney disease [29,30]. To validate our hypothesis that hnRNPK binding FLIP in NLRP3 inflammasome activation, we first prepared a peripheral inflammation model with LPS-stimulated Raw264.7 macrophages to investigate whether hnRNPK participates in activation of inflammatory macrophages by regulating cellular inflammation reaction. Moreover, whether FLIP was involved in the regulation of hnRNPK in NLRP3 inflammasome activation was further determined.

## Materials and methods

### Cell culture and treatment

Raw264.7 macrophages were obtained from the American Type Culture Collection (ATCC, Rockville, MD, USA) and cultured in RPMI-1640 medium (Gibco) with 10% fetal bovine serum (FBS, Gibco) in a humidified incubator containing 5% CO_2_ at 37°C. For constructing inflammasome, RAW264.7 macrophages were treated with 20 ng/mL LPS (Sigma) for 4 h, followed by 5 nM ATP (Sigma) incubation for 30 min. RAW264.7 macrophages were divided into four groups: Control, LPS, ATP and LPS + ATP.

### ELISA

ELISA assay was performed to detect the levels of cytokines released from Raw264.7 macrophages from four groups. In brief, the supernatants were collected and the expression of TNF-α, IL-1β and IL-18 were determined with their respective ELISA kits according to the manufacturer’s instructions (Elabscience). According to the drawn standard curves, the concentration of TNF-α, IL-1β and IL-18 were calculated.

### Western blot

Raw264.7 macrophages were washed with phosphate buffer saline (PBS) and lysed with RIPA (Biosharp) for 30 min. A total of 30 μg protein was separated by 10% SDS-PAGE and electro-transferred to polyvinylidene fluoride (PVDF) membranes (Millipore). The membranes were blocked in TBS with 0.2% Tween 20 (TBST) containing 5% (W/V) dried skimmed milk for 1 h at room temperature. Then, the membranes were incubated with primary antibodies against hnRNPK, FLIP, NLRP3, pro Caspase-1, Caspase-1, pro IL-1β, IL-1β, pro IL-18, IL-18 and GAPDH (Abcam) overnight, followed by incubation with corresponding HRP-conjugated secondary antibodies. The membranes were subsequently exposed to enhanced ECL chemiluminescent substrate (Beyotime Biotech). GAPDH was used as an internal control.

### Cell immunofluorescence

RAW264.7 macrophages were fixed with 4% paraformaldehyde for 15 min and permeated with 0.3% Triton X-100 for 15 min at room temperature. After washed with PBS for 1 h, the cells were incubated with primary antibodies against hnRNPK, FLIP and NLRP3 (Abcam) at 4 °C overnight. The coverslips were exposed to Alexa Fluor conjugated-secondary antibodies (Cell Signaling Technology) for 1 h in the dark. The nucleus was marked with 4′, 6-Diamidino-2-phenylindole (DAPI). The stained images were observed using an Inverted/Fluorescence Microscope (LIONHEART ^LX^, BioTek).

### Cell transfection

Three different small interfering RNA sequences targeting hnRNPK (sihnRNPK-1: 5′-GAGGAAUAAUUGGUGUUAAUU-3′, sihnRNPK-2: 5′-GGGAGAUCUAAUGGCUUAUUU-3′ and sihnRNPK-3: 5′-GCCCAUCAGAAUGGCAAAUUU-3′), three different siRNA targeting FLIP (siFLIP-1: 5′-CCUCCUGGAUAGCUUAAGUUU-3′, siFLIP-2: 5′-CACCUGGUUUCUGAUUAUAUU-3′ and siFLIP-3: 5′-GGCCCAACAUCAAGACUAUUU-3′) and negative control siRNA (si-NC: 5′-UUCUCCGAACGUGUCACGUTT-3′) were synthesized by RiboBio (Guangzhou, China). Knockdown of hnRNPK or FLIP was achieved using Lipofectamine 2000 (Invitrogen) according to the manufacturer’s instructions. Briefly, RAW264.7 macrophages were cultured in 6-well plates at 37 °C overnight and transfected with different sihnRNPK or siFLIP at a final concentration of 100 nM for 48 h, followed by LPS and ATP treatment. Subsequently, the macrophages were collected for the *in vitro* experiments.

### Real time PCR

Total RNA was extracted from macrophages using Trizol reagent (TAKARA). After RNA quantification using spectrophotometry, cDNA was synthesized using a Reverse Aid first strand cDNA synthesis kit (DBI Bioscience). Quantitative real time PCR analysis was performed on Stratagene Real-Time System (AgilentA) using SYBR Green PCR reagent kit (DBI Bioscience). The specific primer sequences were as follows: hnRNPK: forward 5′-CAGCTCCCGCTCGAATCTG-3′ and reverse 5′-ACCCTATCAGGTTTTCCTCCAA-3′; FLIP: forward 5′-TTACACAGGCAGAGGCAAGA-3′ and reverse 5′-GCTGGACTGGGTGTACTTCT-3′; NLRP3: forward 5′-GTGTTGTCAGGATCTCGCAT-3′ and reverse 5′-CTGCAAGTTACACTGTGGGT-3′; Caspase-1: forward 5′-CATCTTTCTCCGAGGGTTGG-3′ reverse 5′-TGTGGTCCCACATATTCCCT-3′; IL-1β: forward 5′-AATGCCACCTTTTGACAGTGATG-3′ and reverse 5′-TGTGCTGCTGCGAGATTTG-3′; IL-18: forward 5′-GACTCTTGCGTCAACTTCAAGG-3′ and reverse 5′-CAGGCTGTCTTTTGTCAACGA-3′; β-actin: forward 5′-CATTGCTGACAGGATGCAGA-3′ and reverse 5′-CTGCTGGAAGGTGGACAGTGA-3′. The gene expression was quantified using 2^−ΔΔCT^ method with β-actin as internal control.

### Co-immunoprecipitation (Co-IP)

Endogenous Co-IP was performed to validate the interaction between hnRNPK and FLIP. Briefly, harvested macrophages from control, LPS + ATP + si-NC or LPS + ATP + si-hnRNPK groups were dealt with lysis buffer. Total protein was incubated with specific antibodies against FLIP on shaking tables, followed by incubation with protein G-sepharose beads (Thermo Fisher Scientific, Waltham, MA, USA). Then, the immunoprecipitated samples were washed with pre-cooling PBS for three times (5 min each time), followed by western blotting as described previously [31].

### Statistical analysis

All experiments were repeated three times. Statistical analysis was performed using SPSS version 20.0 (SPSS Inc., Chicago, IL, USA). Quantitative data were presented as the means ± standard deviation (SD). Differences between two groups were analyzed using an unpaired *t* test and among groups were assessed by one-way analysis of variance (ANOVA) with Shapiro-Wilk test. The results were considered to be statistically significant when *p* values less than 0.05.

## Results

### hnRNPK and FLIP were significantly up-regulated in LPS + ATP-activated NLRP3 inflammasome of macrophages

**Figure 1.**
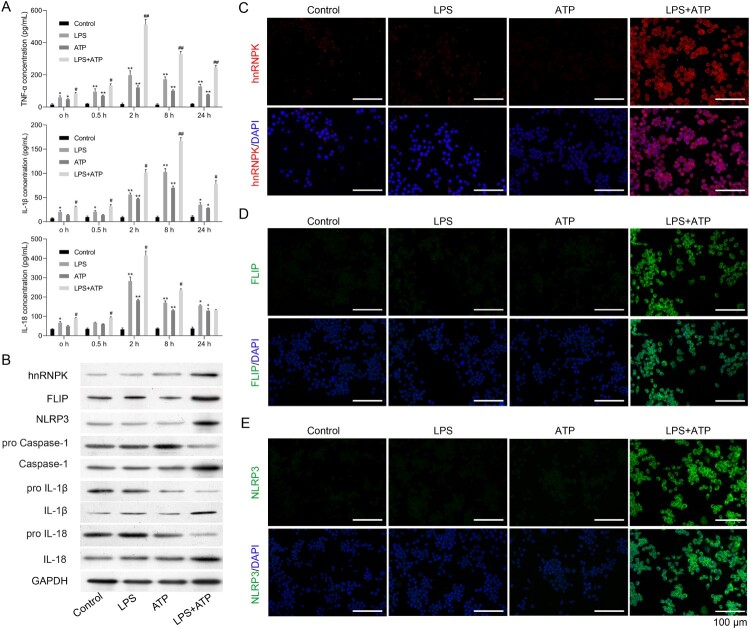
hnRNPK and FLIP were significantly up-regulated in LPS + ATP-activated NLRP3 inflammasome of macrophages. RAW264.7 macrophages were incubated with LPS, followed by ATP incubation (A) Inflammatory factors TNF-α, IL-1β and IL-18 expressions were measured at different time points via ELISA. *p < 0.05, **p < 0.01 *vs.* Control; ^#^*p* < 0.05, ^##^*p* < 0.01 *vs.* LPS; (B) The protein expressions of hnRNPK, FLIP, NLRP3, pro Caspase-1, Caspase-1, pro IL-1β, IL-1β, pro IL-18 and IL-18 in different groups were evaluated by western blot. The expression and distribution of (C) hnRNPK, (D) FLIP and (E) NLRP3 were determined in four groups were detected via immunofluorescence.

To investigate the role of hnRNPK in NLRP3 inflammasome activation associated with CDK progression, macrophage inflammasome was constructed in RAW264.7 macrophages by LPS with ATP treatment. ELISA assay indicated that LPS single stimulation raised the TNF-α, IL-1β and IL-18 expressions in supernatant of RAW264.7 macrophages (*p* < 0.05, *p* < 0.01). ATP single treatment also enhanced the TNF-α, IL-1β and IL-18 expressions in supernatant of RAW264.7 macrophages (*p* < 0.05, *p* < 0.01). But the effects of ATP treatment on TNF-α, IL-1β and IL-18 in RAW264.7 macrophages were weaker than LPS stimulation. ATP + LPS treatment induced a rapid release of TNF-α, IL-1β and IL-18 at different time points (Figure 1A, *p* < 0.05, *p* < 0.01). Moreover, the results of western blot displayed that the protein levels of hnRNPK, FLIP and NLRP3 in ATP treatment of LPS-primed macrophages was obviously up-regulated compared with LPS or ATP single group (Figure 1B). The protein expressions of Caspase-1, IL-1β and IL-18 were also remarkably raised in ATP treatment of LPS-primed macrophages, which were accompanied with the decreased expressions of pro-Caspase-1, pro-IL-1β and pro-IL-18. Furthermore, similar results were found in immunofluorescence, which showed that the expression levels of hnRNPK, NLRP3 and FLIP were all elevated after stimulated by LPS+ ATP treatment (Figure 1C–E).

### hnRNPK knockdown significantly suppressed NLRP3 inflammasome activation

**Figure 2.**
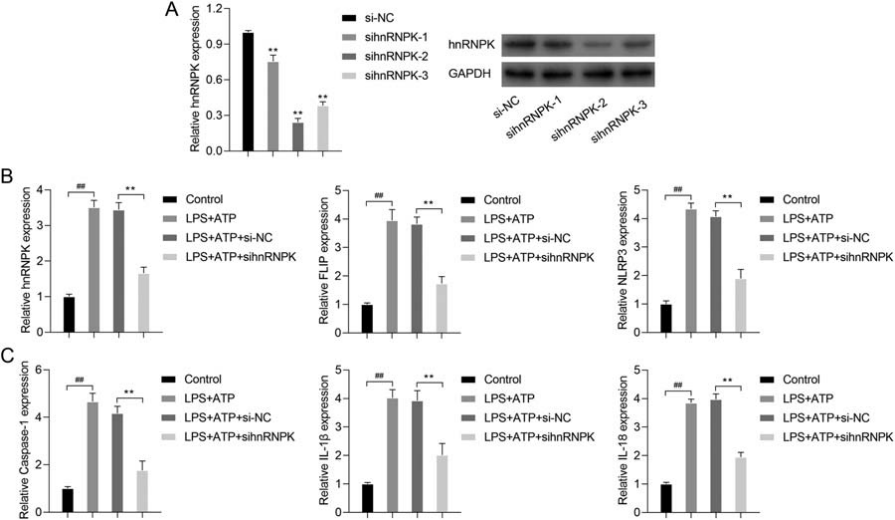
hnRNPK knockdown decreased the expressions of FLIP, NLRP3, Caspase-1, IL-1β and IL-18 in LPS + ATP-activated NLRP3 inflammasome. (A) RAW264.7 macrophages were transfected with three different siRNAs targeting hnRNPK, followed by incubation with LPS and ATP, the mRNA and protein expressions of hnRNPK were detected by real-time PCR and western blot. **p < 0.01 *vs.* si-NC. RAW264.7 macrophages were transfected with sihnRNPK-2, followed by incubation with LPS and ATP, the mRNA expressions of hnRNPK, FLIP, NLRP3 (B), as well as Caspase-1, IL-1β, IL-18 (C) were detected by real-time PCR. ^##^*p* < 0.01 *vs.* Control, **p < 0.01 *vs.* LPS + ATP + si-NC.

**Figure 3.**
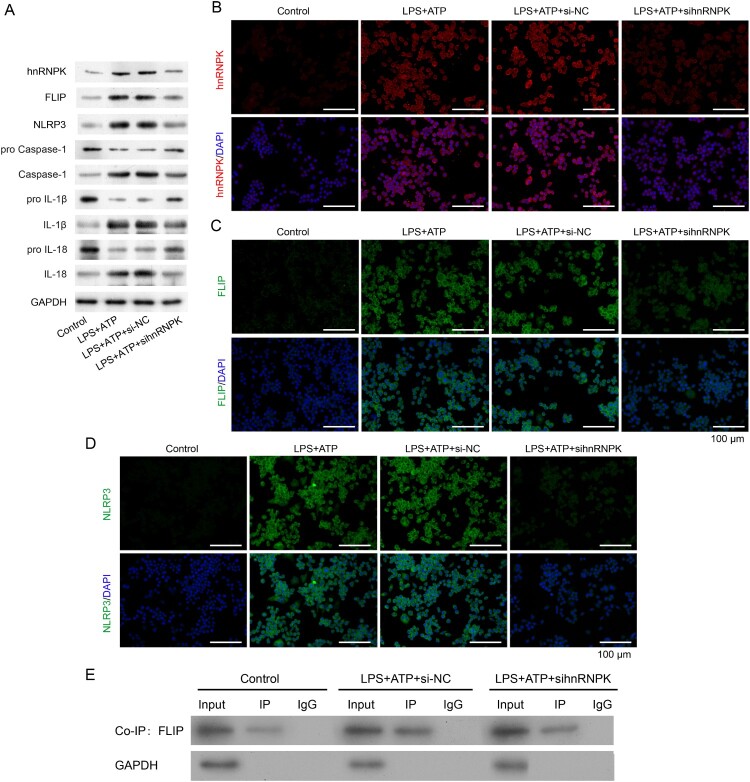
hnRNPK knockdown decreased activated NLRP3 inflammasome related protein levels and bound to FLIP. RAW264.7 macrophages were transfected with si-hnRNPK, followed by incubation with LPS and ATP. (A) The protein expressions of hnRNPK, FLIP, NLRP3, pro Caspase-1, Caspase-1, pro IL-1β, IL-1β, pro IL-18 and IL-18 were evaluated by western blot. The expression and distribution of (B) hnRNPK, (C) FLIP and (D) NLRP3 were determined via immunofluorescence. (E) The binding relationship between hnRNPK and FLIP were detected by co-IP.

Since hnRNPK was up-regulated in LPS + ATP-activated NLRP3 inflammasome, we thus performed loss-of-function assay in RAW264.7 macrophages. As shown in Figure 2A, three different siRNA sequences were designed to silence hnRNPK. The sihnRNPK-1, sihnRNPK-2 and sihnRNPK-3 significantly suppressed the expression of hnRNPK in LPS+ ATP induced macrophages. Notably, sihnRNPK-2 transfection produced the most obvious inhibitory effects, thus sihnRNPK-2 was selected for the subsequent experiments. Next, we further detected the effects of hnRNPK on activation of NLRP3 inflammasome. Real time PCR demonstrated that the mRNA levels of hnRNPK, FLIP, NLRP3, Caspase-1, IL-1β and IL-18 were significantly increased in LPS + ATP-induced macrophages compared with control group (*p* < 0.01), but remarkably decreased after hnRNPK knockdown (*p* < 0.01) (Figure 2B–C). The western blot (Figure 3A) and immunofluorescence analysis of hnRNPK (Figure 3B), FLIP (Figure 3C) and NLRP3 (Figure 3D) also confirmed a significant decrease of hnRNPK, FLIP and NLRP3 expressions in the hnRNPK silencing NLRP3 inflammasome. Besides, after hnRNPK knockdown, the protein levels of Caspase-1, IL-1β and IL-18 were also reduced in LPS + ATP-induced macrophages (Figure 3A).

### hnRNPK binds to FLIP in LPS + ATP-activated NLRP3 inflammasome

The above results showed that FLIP was down-regulated after hnRNPK knockdown, we thus speculated that FLIP might interact with hnRNPK. To confirm our hypothesis, hnRNPK-FLIP binding was detected by co-IP in LPS + ATP-activated NLRP3 inflammasome. As depicted in Figure 3E, the interaction of FLIP and hnRNPK was weakened after hnRNPK knockdown in LPS + ATP-activated NLRP3 inflammasome. These results suggest that hnRNPK might affect the activation of NLRP3 inflammasome by directly interacting with FLIP.

### FLIP knockdown imitated the effects of hnRNPK silencing on the activation of NLRP3 inflammasome

**Figure 4.**
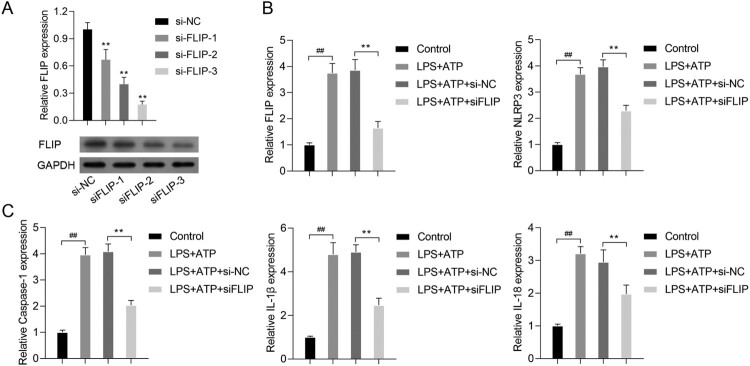
FLIP knockdown decreased the expressions of FLIP, NLRP3, Caspase-1, IL-1β and IL-18 in LPS + ATP-activated NLRP3 inflammasome. (A) RAW264.7 macrophages were transfected with three different siRNA targeting FLIP, followed by incubation with LPS and ATP. the mRNA and protein expressions of FLIP were detected by real-time PCR and western blot. **p < 0.01*vs.* si-NC. RAW264.7 macrophages were transfected with si-FLIP-3, followed by incubation with LPS and ATP, the mRNA expressions of FLIP, NLRP3 (B), as well as Caspase-1, IL-1β, IL-18 (C) were detected by real-time PCR. ^##^*p* < 0.01 *vs.* Control, **p < 0.01 *vs.* LPS + ATP + si-NC.

**Figure 5.**
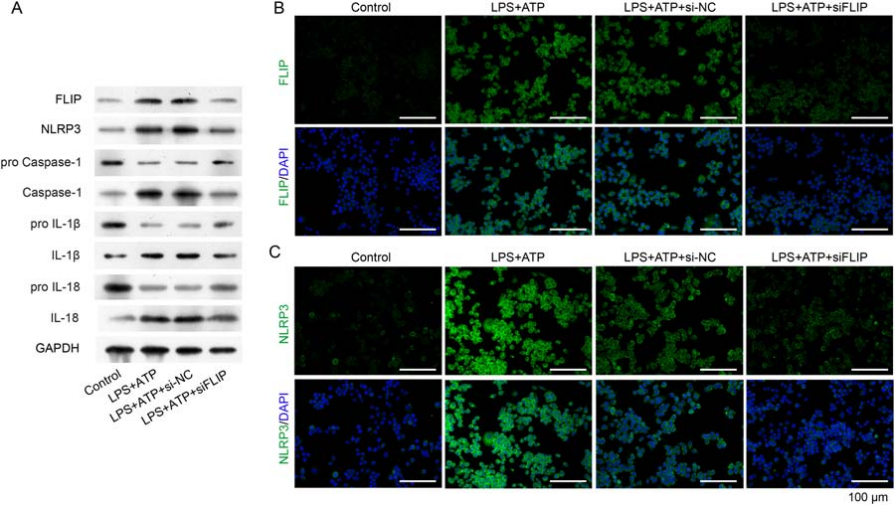
FLIP knockdown decreased activated NLRP3 inflammasome related protein levels. RAW264.7 macrophages were transfected with si-FLIP, followed by incubation with LPS and ATP. (A) The protein expressions of FLIP, NLRP3, pro Caspase-1, Caspase-1, pro IL-1β, IL-1β, pro IL-18 and IL-18 were measured by western blot. (B–C) The expression and distribution of FLIP and NLRP3 were detected by immunofluorescence staining assay.

Our previous study showed that FLIP was positively correlated with hnRNPK (data not shown). Based on the physical interaction between FLIP and hnRNPK, we further investigated the biological role of FILP in LPS + ATP-activated NLRP3 inflammasome. First, we designed three siRNA sequences to specific silence FLIP in RAW264.7 macrophages, as confirmed by real time PCR and western blot analysis (Figure 4A, *p* < 0.01). It was worthy noted that siFLIP-3 generated the strongest silencing effects on FLIP expression in all the three siRNA sequences (*p* < 0.01), which was utilized for the subsequent analysis. Consistently with hnRNPK knockdown, silencing FLIP significantly down-regulated the expression of FLIP, NLRP3 and Caspase-1, as well as the inflammatory factors (IL-1β and IL-18), as determined by, real time PCR (Figure 4B–C, *p* < 0.01), western blot (Figure 5A) and immunofluorescence (Figure 5B and C), respectively. These results demonstrated that FLIP might be involved in the regulatory effect of hnRNPK on NLRP3 inflammasome activation.

## Discussion

Increasing evidences show that the endotoxin model using LPS has been widely applied to explore the regulatory mechanism of the inflammatory response in several diseases, including acute lung injury [32] and neurodegenerative diseases [33], as well as CKD [34]. Hwang et al. [35] reported that LPS-stimulated Raw264.7 macrophages accelerated pro-inflammatory and pro-fibrotic cytokines, which contributed to accelerate chronic kidney diseases. Nagase et al. [36] indicated that LPS-stimulated Raw264.7 could be serving as an *in vitro* cell model to simulate renal inflammation and injury. In the present study, *in vitro* LPS-stimulated mouse RAW264.7 macrophages was used to investigate the regulatory mechanism of hnRNPK and FLIP in NLRP3 inflammasome activation. We found that LPS induced the production of TNF-α, IL-1β and IL-18 in the culture medium of RAW264.7 macrophages. Moreover, LPS plus ATP activated the NLRP3 inflammasome, reflected by up-regulation of TNF-α, IL-1β and IL-18. Consistently, NLRP3 inflammasome consists of NRLP3 and caspase-1 which is implicated in the pathogenesis of inflammatory diseases by elevating the secretion of pro-inflammatory cytokines [37–39]. In addition, loss of NLRP3 significantly reduced inflammation and tubulointerstitial fibrosis in mice after unilateral ureteral obstruction [40]. Herein, we found that both the NLRP3 and Caspase-1 expressions were increased after LPS plus ATP treatment, which further confirmed the activation of NLRP3 inflammasome.

Previous studies have demonstrated that hnRNPK directly related to proliferation, migration and autophagy in several diseases [41–43]. However, it remains unclear whether hnRNPK plays an important role in NLRP3 inflammasome in LPS induced RAW264.7 macrophages. In our study, we found that hnRNPK was significantly up-regulated in activated NLRP3 inflammasome. Knockdown of hnRNPK remarkably alleviated LPS + ATP-induced increased expressions of NLRP3, Caspase-1, IL-1β and IL-18. Bomsztyk et al. [18] has demonstrated that hnRNPK localizes in both the nucleus and cytoplasm and preferentially recognizes poly-C sequences of target RNAs through its three repeats of K homology domains (KH1-3), which play a central role in many fundamental cellular processes, such as cell proliferation, apoptosis and differentiation [44,45]. Similar to our results, Lichtnekert et al. [23] found that hnRNPK contributed to the activation of macrophages by up-regulating the transcription of TNF-α, IL-1A, IL-1B and IL-10. These findings suggested that hnRNPK was closely related to the activation of NLRP3 inflammasome in LPS + ATP-stimulated mouse RAW264.7 macrophages and might play regulatory role in the progression of CKD.

Notably, co-IP assay further showed that hnRNPK directly interacts with FLIP in LPS + ATP-stimulated mouse RAW264.7 macrophages. Moreover, FLIP knockdown imitated the effects of hnRNPK knockdown on the expression of NLRP3, Caspase-1, IL-1β and IL-18 in LPS + ATP-stimulated mouse RAW264.7 macrophages. These was consistent with the previous study, which showed that reduction of FLIP in macrophages could selectively suppress inflammation in arthritis [25]. Taken together, these findings suggested that the connection of hnRNPK-FLIP induced the activation of NLRP3 inflammasome in LPS + ATP-stimulated mouse RAW264.7 macrophages via prompting Caspase-1-medicated IL-1β and IL-18 activation (Graphical abstract).

In conclusion, our study shows that hnRNPK directly binds FLIP to regulate the NLRP3 inflammasome activation in response to LPS + ATP stimulation, which might be a potential regulatory mechanism underlying the progression to CKD.

## Author’s contribution statement

Junxia Feng, Hongyan Li, Wei Sun and Yunfang Zhang: Conception and design, financial support, manuscript writing. Jingchun Li and Ping Meng: Administrative support, Collection and assembly of data. Lina Wang, Chunli Liu and Shili Zhao: Data analysis and data interpretation. All authors read and approved the final manuscript.

## Data Availability

The authors confirm that the data supporting the findings of this study are available within the article.
